# Loneliness shapes disparities in healthy life expectancy: a multi-state analysis from China

**DOI:** 10.1186/s12889-024-18975-z

**Published:** 2024-06-04

**Authors:** Qi Yu, Yiting Ren, Jilei Wu

**Affiliations:** 1 Institute of Population Research, Peking University, Beijing, China; 2 Department of Mathematics, University College London, London, UK

**Keywords:** Older adults, Loneliness, Healthy life expectancy, Gender differences, Activity of daily living, Self-rated health

## Abstract

**Objectives:**

To assess the influence of loneliness on the healthy life expectancy of older adults in China and its gender disparities across different health indicators, in order to provide insights for enhancing the health status and subjective well-being of the older population.

**Method:**

We conducted a cohort analysis using four waves of weighted samples (2008, 2011, 2014, and 2018) from the Chinese Longitudinal Healthy Longevity Survey, encompassing 15,507 respondents aged 65–99. Physical and subjective health were assessed through activity of daily living (ADL) and self-rated health (SRH), respectively. Utilizing loneliness status as a time-variant variable, we employed the multi-state interpolated Markov Chain to explore the associations between loneliness and age-specific life expectancy (LE), healthy life expectancy (HLE), and the proportion of healthy life expectancy in life expectancy (HLE/LE).

**Results:**

Compared to the non-lonely population, both LE and HLE were lower among lonely individuals. Regarding gender differences, the HLE/LE for females in the lonely population was consistently lower than that for males. The impact of loneliness on the health of older adults varied by measurement indicators and gender. Specifically, based on ADL results, the decline in HLE/LE was greater for females, with a decline of 53.6% for lonely females compared to 51.7% for non-lonely females between the ages of 65 and 99. For males, the decline was 51.4% for lonely males and 51.5% for non-lonely males. According to SRH, the gender difference in the decline of HLE/LE due to loneliness was less apparent. For males, the change in HLE/LE for non-lonely individuals was 3.4%, compared to 4.2% for lonely individuals, whereas for females, the change was 3.7% for non-lonely individuals and 4.4% for lonely individuals.

**Conclusion:**

Loneliness exerts varied effects on health across different measurement indicators and gender demographics. Targeted health promotion interventions are imperative to mitigate these negative impacts, particularly emphasizing the enhancement of subjective well-being and physical functioning, especially among older adult females.

**Supplementary Information:**

The online version contains supplementary material available at 10.1186/s12889-024-18975-z.

## Introduction

Loneliness is a world-spread social and public health concern [1]. This negative emotional state signifies a sense of mental disconnect and dissatisfaction with relationships [2–5]. Research on loneliness predominantly emphasizes developed nations, where 20%-40% of older adults experience loneliness [6].

Although family values and social support systems are highly valued in China, urbanization and changes in family structure have led to decreased intimacy and mutual support between older adults and their children, increasing the risk of loneliness among older adults. Especially as China's population continues to age and with limited social resources, the necessary social support and attention for older adults may be insufficient, leading to a growing prevalence of loneliness among them. It has been reported that approximately one-fourth of older adults in China report feeling lonely [7, 8]. For example, during the Corona Virus Disease 2019 (COVID-19) pandemic, quarantine measures and social distancing have isolated many older adults from their families and communities, further increasing their risk of loneliness [9]. Therefore, understanding the issue of loneliness among older adults in China is crucial for formulating relevant policies and providing support services.

Loneliness can lead to adverse health consequences with multiple mechanisms [10–12]. For example, loneliness acts as a stressor to trigger reactions in autonomic nervous system, including the hypothalamic–pituitary–adrenal axis (HPAA) and autonomic nervous system [13], which had been approved in biological mechanisms. Additionally, gender differences are inherent social characteristics, and these disparities contribute to variations in the health implications of loneliness. This discrepancy significantly influences the coping mechanisms employed by individuals and others respond to loneliness [10], affecting health behaviors, social interactions, and healthcare access [14].

Loneliness is closely linked to numerous detrimental health outcomes, encompassing both physical and subjective well-being. This association is particularly pronounced among older adults, due to changing social roles and the heightened risk of age-related diseases [15]. Substantial research provides evidence supporting these connections, including associations with cardiovascular diseases [16], physical limitations [17], cognitive impairment [18], and poor self-rated health [19]. However, many studies have predominantly examined specific health outcomes without considering the broader spectrum of health indicators. It is crucial to account for the multidimensional aspects of health and well-being when investigating the influence of loneliness on overall health. For instance, incorporating measures like self-rated health (SRH) for subjective well-being and activity of daily living (ADL) / instrumental activity of daily living (IADL) for physical health assessment can provide a more comprehensive understanding.

Furthermore, there is a limited amount of research that directly compares the extent of loneliness impact on physical and subjective health. Many studies primarily rely on prevalence rates, often neglecting the implications of mortality selection and standardized age and sex distributions. The application of healthy life expectancy (HLE) can effectively mitigate the limitations associated with prevalence rates. For instance, a notable study conducted in Singapore employed SRH, ADL, and IADL to calculate HLE and investigate the influence of loneliness on both physical and subjective indicators. Nonetheless, this study did not directly compare the relative effects of loneliness on these distinct dimensions of health. Moreover, these investigations are notably scarce in developing nations, where the challenges of aging are increasingly prominent within the realm of social development.

Additionally, the distribution of loneliness among older people and its impact on health exhibits gender-related variations. Older women are more susceptible to experiencing loneliness and spreading more rapidly than men [7, 10, 20]. Researches have demonstrated that loneliness affects older women more profoundly than men in relation to physical function [21, 22], depression [23], and cognitive impairment [18]. However, the results are contradictory concerning mortality [24, 25]. Given the inconsistencies in existing literature regarding gender differences, we attribute this by two aspects. Firstly, the measurement of loneliness is complex as it fluctuates with health status and social circumstances of respondents. Secondly, population health studies frequently grapple with mortality selection and often necessitate the standardization of prevalence and mortality according to age and sex. Therefore, we treat loneliness as a time-varying variable and analyze its relationship with health using the HLE framework.

By excluding age structure and integrating both mortality and morbidity, HLE serves as a reliable assessment tool for evaluating the quality of life, utilizing ADL for physical health and SRH for subjective health. Employing a multi-state Markov chain model, we treat loneliness as a timing variable to ascertain its ongoing impact on health during old age, while also exploring gender differences and various health indicators. Notably, while developed nations have adopted strategies to prevent or alleviate loneliness among older adults, this issue has yet to receive adequate attention in developing countries [11]. Given the aging population, declining fertility rates, and increased population mobility, addressing the loneliness of older adults is poised to become a pressing public and social concern. Consequently, our study aims to raise awareness about the emotional well-being of older adults, assist policymakers in pinpointing target demographics for targeted interventions against loneliness, and effectively enhance the health status and quality of life for older adults.

## Methods

### Data

This cohort study examined data from four waves (2008, 2011, 2014, and 2018) of the Chinese Longitudinal Healthy Longevity Survey (CLHLS), a nationally representative longitudinal study jointly conducted by Peking University and Duke University. The baseline wave encompassed 16,954 respondents aged 65 and older from 23 provinces. Its purpose was to analyze the health and longevity of Chinese older adults and factors impacting them. The study employed a multistage disproportionate and targeted random sampling method to uphold sample representativeness, supplemented by baseline survey weights [26, 27].

We ultimately included an eligible sample of 15,507 individuals with weighting, all of whom met the following criteria at baseline: (1) absence of residence in care facilities, (2) age between 65 and 99 years, (3) engagement in the survey and follow-up for at least one wave, and (4) precise assessment of ADL and SRH. The subsequent survey waves comprised 13,200 participants in 2011, 12,526 in 2014, and 10,304 in 2018. Measurements were longitudinally collected from 2008 to 2018 for each respondent. All analyses were weighted for national representativeness and compared to the sixth Census data in China (Refer to supplementary material Figure S1 for conciseness).

### Measurements

#### Mortality

The CLHLS collected death information in each wave, with family members providing death dates. Research has validated the alignment of age-sex-specific mortality with the Kannisto model [28], renowned for its accurate representation of mortality patterns, particularly in aging populations. Developed by Finnish statistician Lauri Kannisto, this statistical tool predicts population mortality rates and life expectancy by relying on historical mortality rate variations to forecast future trends. Its insights are invaluable for analyzing population aging and guiding policy decisions [29].

### Loneliness

Loneliness can be assessed through two main methods: one-item measures and multi-item measures [2]. One-item measures directly inquire about the frequency or intensity of loneliness, while multi-item measures encompass scales like the UCLA Loneliness Scale, DeJong-Gierveld’s Loneliness Scale, and Ryff’s Scale of social integration [29]. While one-item measures save time and screening barriers, multi-item measures offer a comprehensive assessment of loneliness [30]. The one-item approach demonstrates strong construct validity and is highly aligned with subjective health, encompassing well-being, depression, and SRH. Furthermore, it exhibits high correlation with multi-item measures, enabling comprehensive and multidimensional assessment of social determinants of health [29, 30].

Loneliness was considered a time-varying variable from 2008 to 2018 and was evaluated using the one-item measures within the CLHLS. Participants were asked the question, “Do you often feel lonely and isolated?”, with options including “always”, “often”, “sometimes”, “seldom”, and “never”. Those who responded “sometimes” or higher were classified as feeling lonely, whereas those selecting “seldom” or “never” were categorized as non-lonely.

### Health status: ADL & SRH

Both ADL and SRH were considered time-varying variables, derived from each wave. ADL served to evaluate physical health, employing the Katz Activities of Daily Living Scale, a well-established and reliable method encompassing six items: eating, dressing, using the toilet, indoor transfers, continence, and bathing [31]. To be classified as having no activity limitation, respondents needed to complete all ADLs independently; otherwise, they were categorized as having activity limitation.

SRH is globally recognized as an indicator of subjective health. It is frequently utilized in sociological surveys and demonstrates strong robustness and reliability, especially in longitudinal studies [32]. In the CLHLS, participants were posed with the question, “In general, how would you describe your health?”, and presented with five options: very good, good, so-so, bad, and very bad. Individuals who selected “good” or “very good” were categorized as healthy, whereas those who chose any other response were grouped within the unhealthy category.

### Outcomes

Using ADL and SRH as metrics, we calculated two types of life expectancy (LE), HLE, and the proportion of healthy life expectancy in life expectancy (HLE/LE).

### Covariates

Demographic and social characteristics that might potentially impact the connection between loneliness and health status were concerned. These characteristics comprised gender, residence, education, income, marital status, living arrangements, and region. These factors were maintained as constant variables and documented during the baseline survey.

Gender was employed for classification and comparison purposes. Residence was categorized as rural and urban (encompassing cities and towns). Educational level was divided into two groups based on years of schooling: uneducated and educated. Income classification consisted of high income and low income, determined by individual self-assessment. Marital status was dichotomized into spouse and no spouse. The no spouse category encompassed individuals who were married but not living with spouse, divorced, widowed, and never married. Living arrangements were bifurcated into living alone and living with others, excluding those not residing in institutions. Geographical regions were categorized as eastern, central, and western, following criteria set by the National Bureau of Statistics of China.

### Statistical analysis

The study employed independent sample *t* tests and Chi-square tests to analyze gender differences in demographic characteristics and health status. With loneliness and health status as timing variables, population-based LE and HLE were calculated by gender using the multi-state life table of interpolated Markov chain (iMach). This approach relies on longitudinal survey data, enabling mutual transitions between health states and treating death as an absorbing state. By employing interpolation or extrapolation techniques, it estimates the health status of participants lost to follow-up during the survey period. Consequently, the outcomes derived from this approach are more precise compared to those through cross-sectional analysis [33].

**Fig. 1:**
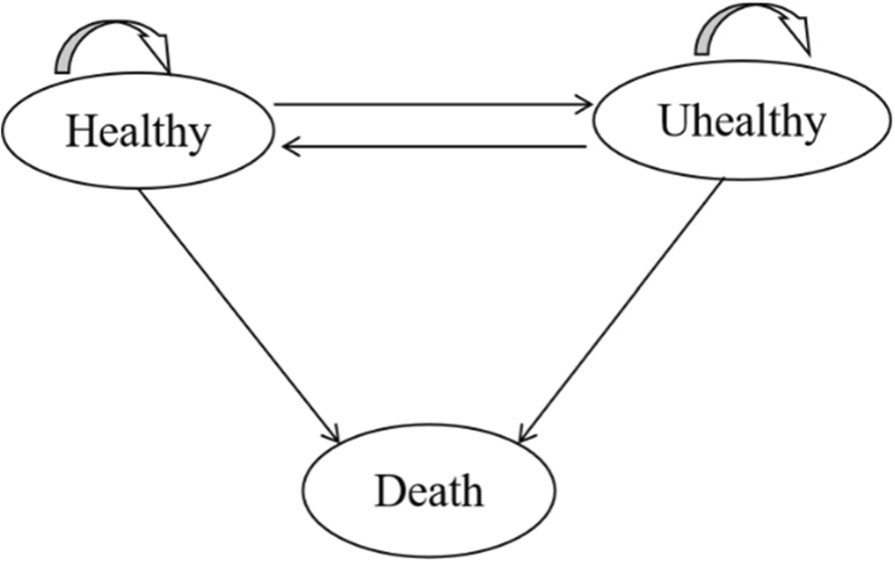
States and possible transitions across states

This analysis involved three health states in each wave: healthy (status 1), unhealthy (status 2), and death (status 3). Inter-transitions between healthy and unhealthy were possible, while death was an absorbing state. Figure 1 illustrates six potential transitions during each interview: from healthy to healthy (p_S1→S1_), from healthy to unhealthy (p_S1→S2_), from healthy to death (p_S1→S3_), from unhealthy to healthy (p_S2→S1_), from unhealthy to unhealthy (p_S2→S2_), and from unhealthy to death (p_S2→S3_).

Based on this, one-year transition probabilities, population-based LE, and HLE were derived. The main formulas are shown below [33].

Let $h$ be the time interval, and assume that the change of individual loneliness status follows the Markov chain during age $(x,x + h)$, then the transition probabilities from $j$ state at age $x$ to $k$ state at age $(x + h)$ is

$$_{h} p_{{^{x} }}^{jk} = \Pr [X(x + h) = k\left| {X(x)} \right. = j]$$

and one step transition matrix:

$$_{h} P_{x} =_{h} p_{{^{x} }}^{jk} = \left( {\begin{array}{*{20}c} {_{h} p_{{^{x} }}^{11} } & {_{h} p_{{^{x} }}^{12} } & {_{h} p_{{^{x} }}^{13} } \\ {_{h} p_{{^{x} }}^{21} } & {_{h} p_{{^{x} }}^{22} } & {_{h} p_{{^{x} }}^{23} } \\ 0 & 0 & 1 \\ \end{array} } \right)$$

If the initial health state is $i$, the stable prevalence rates of the outcome state being 1 (non-lonely) and 2 (lonely) are

$$_{t} w^{i1} (x) = \frac{{_{t} p_{{^{x - t} }}^{i1} }}{{_{t} p_{{^{x - t} }}^{i1} +_{t} p_{{^{x - t} }}^{i2} }}$$

$$_{t} w^{i2} (x) = \frac{{_{t} p_{{^{x - t} }}^{i2} }}{{_{t} p_{{^{x - t} }}^{i1} +_{t} p_{{^{x - t} }}^{i2} }}$$

Let $w^{2} (x,\theta )$ be the ratio of different loneliness conditions at age $x$, regardless of the initial status, the expected survival time for a health outcome $j$ (healthy or unhealthy) is

$$e_{{^{x} }}^{.j} (\theta ) = \left[ {1 - w^{2} (x,\theta )} \right]e_{{^{x} }}^{1j} (\theta ) + w^{2} (x,\theta )e_{{^{x} }}^{2j} (\theta ) = \sum\limits_{y = 0}^{\infty } {{}_{y}p_{{^{x} }}^{.j} \left( {\theta ,w^{2} (x,\theta )} \right)}$$

The HLE at the age $(x,x + y)$ is

$$_{y} e_{{^{x} }}^{ij} = \sum\limits_{u = 1}^{y} {_{u} p_{{^{x} }}^{ij} }$$

The LE at age $x$ is

$$e_{{^{x} }}^{..} = e_{{^{x} }}^{.1} + e_{{^{x} }}^{.2}$$

### Sensitivity analyses

To validate the robustness of the impact of loneliness on health status, this study presented sensitivity analysis results in the supplementary materials. Using the multi-state life table of interpolated Markov chain (iMach), with gender, residence, education, income, marital status, living arrangements, and region as control variables, and loneliness at each survey as a time-varying variable, this study computed LE, HLE, and HLE/LE separately for each gender, using ADL and SRH as measures of health status.

## Results

### Socioeconomic characteristics and health status of older adults

At baseline, the mean age of the 15,507 participants was 72.9 (SD = 6.2), with 7438 men and 8068 women based on weighted data (Table 1). Significant gender disparities were observed across various characteristics. Men had a mean age of 72.50 (SD = 5.89), while women had a mean age of 73.3 (SD = 6.4) years (*p* < 0.001). Concerning socioeconomic attributes, a higher percentage of older women exhibited characteristics compared to men (*p* < 0.001), including uneducated (61.1%), low income (16.5%), absence of a spouse (52.0%), and living alone (17.9%). Loneliness was more prevalent among women (29.5%) than men (20.2%) (*p* < 0.001). In terms of health status, both men (96.0%) and women (96.4%) were more likely to be active, while 82.5% of women self-reported as healthy, significantly lower than men (85.3%) (*p* < 0.001).

**Table 1:** Socioeconomic characteristics and health status of older adults at baseline

Sample characteristic	All (n = 15,507)	Men (*n* = 7438)	Women (*n* = 8068)	Gender difference
	Mean (SD) or %	Mean (SD) or %	Mean (SD) or %	*p* Value
Age	72.93(6.2)	72.50(5.9)	73.32(6.4)	< 0.001
Rural residents	8935 (57.6)	4306(57.9)	4629(57.4)	0.509
Uneducated	6551 (42.2)	1620(21.8)	4930(61.1)	< 0.001
Low income	2399 (15.5)	1071(14.4)	1327(16.5)	< 0.001
No spouse	6051(39.0)	1852(24.9)	4198(52.0)	< 0.001
Living alone	2275 (14.7)	830(11.2)	1445(17.9)	< 0.001
Western area	4448(28.9)	2150(28.9)	2298(28.5)	0.457
Central area	4274(27.6)	2015(27.1)	2258(28.0)	
Feeling lonely	3880 (25.0)	1503(20.2)	2377(29.5)	< 0.001
No activity limitation	14,914 (96.9)	7170(96.0)	7744(96.4)	0.180
Self-rated healthy	13,004 (83.9)	6346(85.3)	6658(82.5)	< 0.001

### Comparing transition probabilities between lonely and non-lonely older adults

Before computing LE and HLE, we calculated one-year transition probabilities (Table S1 and Table S2 in supplementary material) for lonely and non-lonely older adults using ADL and SRH assessments. Lonely individuals exhibited lower probabilities of maintaining good health (p_S1→S1_) and recovering from unhealth(p_S2→S1_) when compared to non-lonely counterparts. Conversely, lonely older adults had higher probabilities of experiencing unhealthy outcomes, including transitioning from health to unhealth (p_S1→S2_), remaining in an unhealthy state(p_S2→S2_), transitioning from health to death (p_S1→S3_), and transitioning from unhealth to death(p_S2→S3_). These patterns were consistent for both ADL and SRH assessments.

### Mortality and health differences between lonely and non-lonely older adults

LE serves as a measure of population mortality. A comparison between non-lonely and lonely individuals revealed significant differences, with the former consistently exhibiting higher LE throughout old age, regardless of whether ADL or SRH was used (Table 2). For instance, considering ADL, non-lonely adults had a LE of 23 years (95% CI: 22.4–23.6) at age 65, surpassing the 19.5 years (95% CI: 18.9–20.0) observed for lonely individuals. Similarly, with respect to SRH, a difference of over 2 years was evident at age 65. Specifically, among lonely older adults, the LE for SRH was 19.9 years (95% CI: 19.3–20.5), compared to 22.2 years (95% CI: 21.8–22.6) for non-lonely individuals. This distinction persisted across other age groups as well.

**Table 2:** Population-based LE, HLE and HLE/LE (%) for lonely and non-lonely older adults by ADL and SRH, with 95% confidence intervals

	ADL			SRH		
Age	LE	HLE	HLE/LE	LE	HLE	HLE/LE
Lonely						
65	19.5 (18.9,20.0)	16.1(15.6,16.7)	82.8	19.9 (19.3,20.5)	15.4(14.8,16.0)	77.4
75	13.0 (12.5,13.4)	9.62(9.2,10.0)	74.0	13.5 (13.0,14.0)	10.6(10.09,11.04)	78.4
85	8.4 (7.9,8.8)	4.9(4.5,5.3)	59.0	8.6 (8.1,9.0)	6.79(6.36,7.22)	79.4
Non-lonely						
65	23 (22.4,23.6)	18.7(18.3,19.1)	81.2	22.2 (21.8,22.6)	18.3(17.8,18.7)	82.3
75	16.1 (15.4,16.7)	11.6(11.2,12.0)	72.4	15.3 (14.9,15.8)	12.8(12.3,13.2)	83.1
85	10.9 (10.2,11.7)	6.3(5.8,6.8)	57.6	10.0 (9.4,10.4)	8.3(7.9,8.8)	84.0

Notes: LE indicates life expectancy, HLE indicates healthy life expectancy, HLE/LE indicates the proportion of healthy life expectancy in life expectancy, ADL indicates activity of daily living and SRH indicates self-rated health

**Fig. 2:**
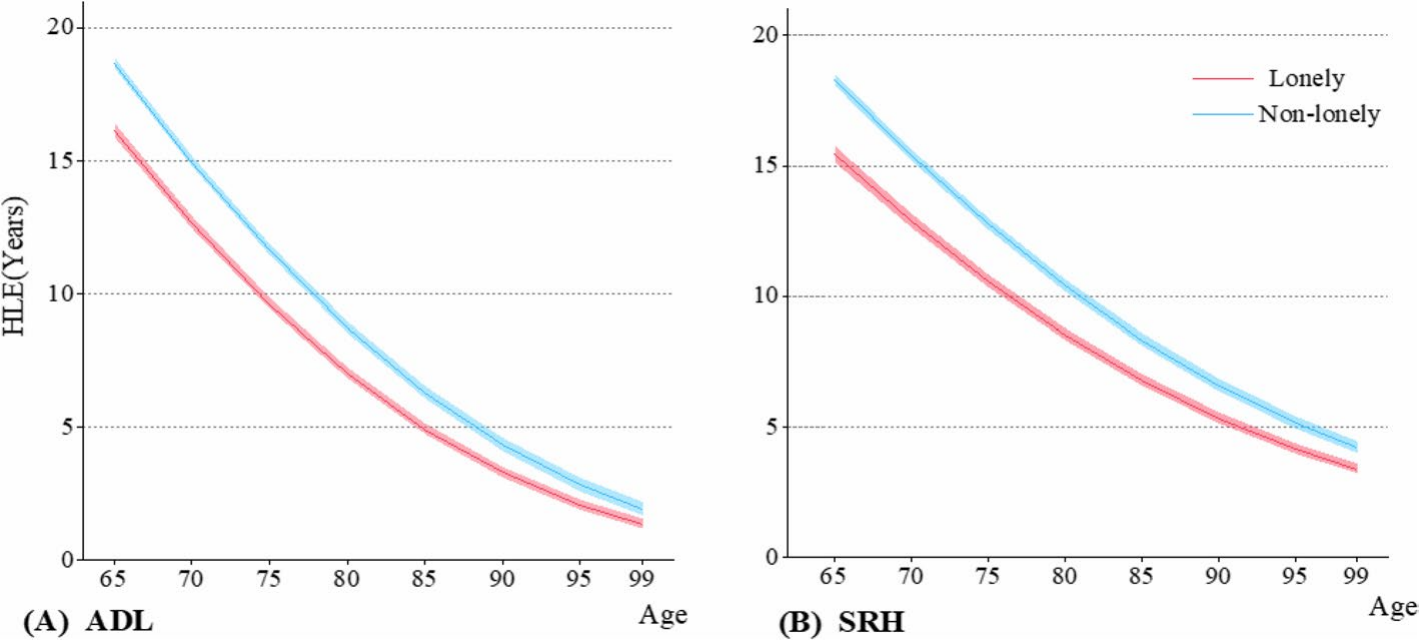
The population-based healthy life expectancy (HLE) for lonely and non-lonely older adults. **A** The healthy life expectancy of lonely calculated by activity of daily living (ADL). **B** The healthy life expectancy of lonely calculated by self-rated health (SRH)

**Fig. 3:**
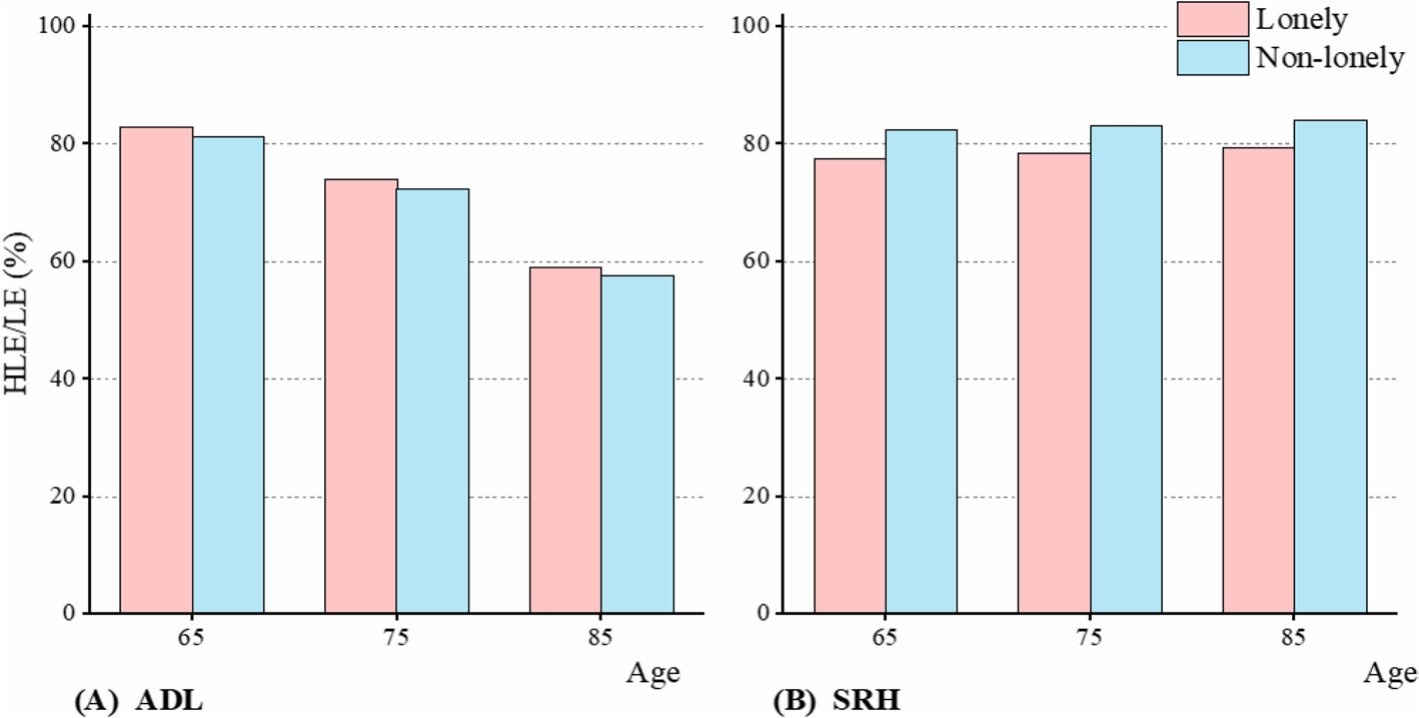
The comparison of HLE/LE for lonely and non-lonely older adults. **A** The HLE/LE calculated by activity of daily living (ADL). **B** The HLE/LE calculated by self-rated health (SRH)

To assess health status and quality of life, we utilized HLE and HLE/LE measures. The findings indicated that lonely older adults exhibited shorter HLE compared to their non-lonely counterparts during advanced aging, whether assessing ALD or SRH (Fig. 2). We compared HLE/LE at ages 65, 75, and 85, highlighting significant disparities between lonely and non-lonely individuals (Fig. 3). Notably, the HLE of lonely older adults were substantially lower than those of non-lonely counterparts, encompassing both physical and subjective health dimensions (Fig. 2). The HLE/LE results vary across different health indicators. Specifically, based on ADL, lonely older adults exhibited a higher HLE/LE, whereas those assessed using SRH showed the opposite trend, with non-lonely older adults having a higher HLE/LE (Fig. 3).

For instance, based on ADL, at age 65, lonely individuals had an HLE of 16.1 years (95% CI: 15.6–16.7), with an HLE/LE of 82.8%, while non-lonely individuals exhibited 18.7 years (95% CI: 18.3–19.1) and 81.2%, respectively (Table 2). Similarly, according to SRH, the HLE for non-lonely individuals at age 65 was 18.3 years (95% CI: 17.8–18.7), almost 3 years longer than lonely older adults who had 15.4 years (95% CI: 14.8–16.0) (Table 2). In terms of HLE/LE, lonely individuals were 77.4%, significantly lower than 82.3% observed in non-lonely older adults (Table 2).

### The association between loneliness and HLE/LE of older adults

**Fig. 4:**
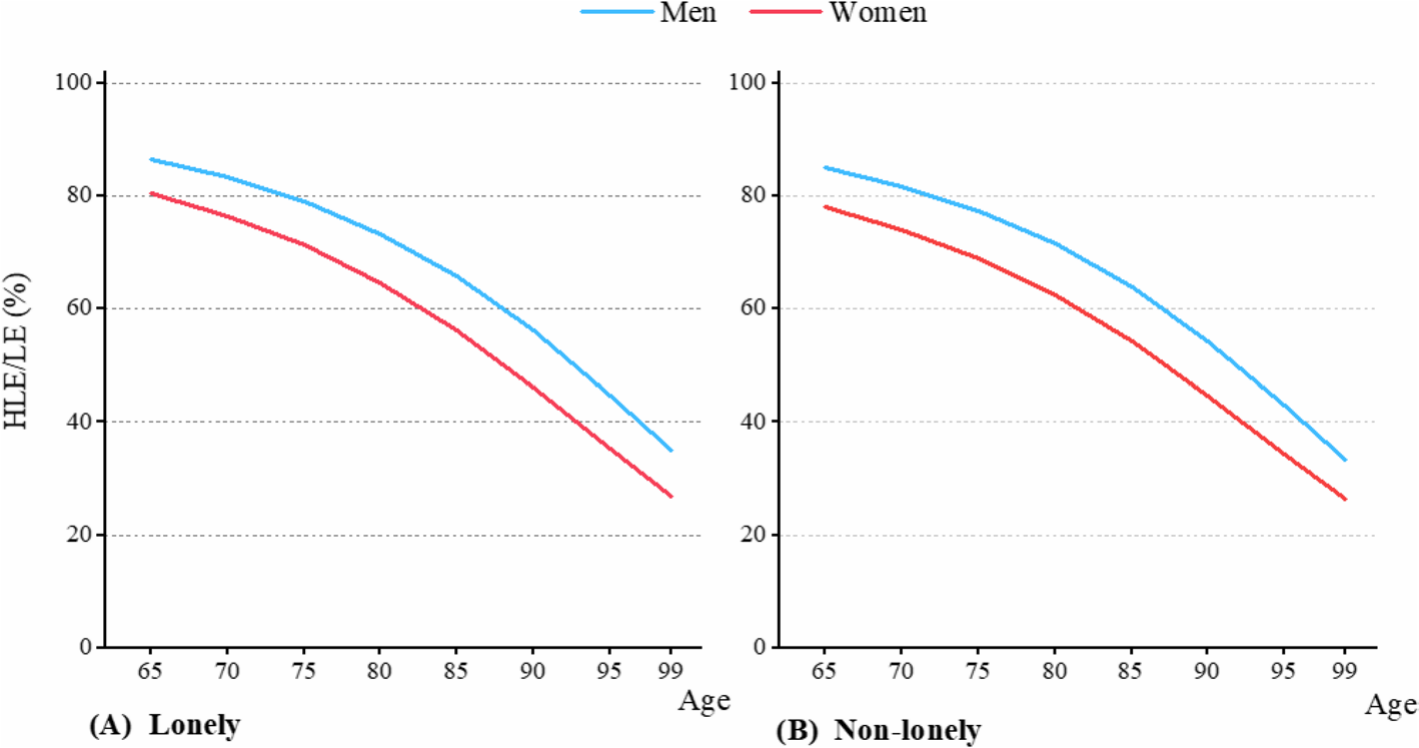
Gender-specific HLE/LE of lonely and non-lonely older adults, based on ADL. **A** The HLE/LE for lonely adults, based on ADL; **B** The HLE/LE for non-lonely adults, based on ADL

**Fig. 5:**
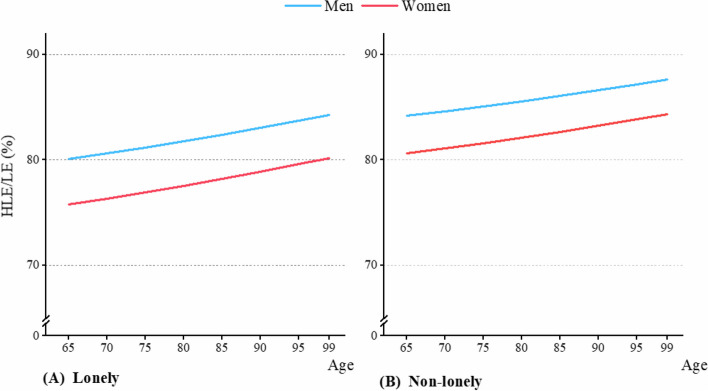
Gender-specific HLE/LE of lonely and non-lonely older adults, based on SRH. **A** The HLE/LE for lonely adults, based on SRH; **B** The HLE/LE for non-lonely adults, based on SRH

According to health indicators, the HLE/LE calculated from ADL showed a rapid decline across the older stage, with lonely older adults exhibiting higher ratio compared to non-lonely older adults (Fig. 4). For instance, among 65-year-old males, the HLE/LE for non-lonely and lonely older adults were 85% and 86.4%, respectively (Table 3). This phenomenon might have been related to living arrangements, as older adults living alone often demonstrated higher levels of self-care ability. According to SRH calculations, HLE/LE showed a slight increase, but lonely older adults had lower HLE/LE compared to non-lonely ones (Fig. 5). For example, among 65-year-old males, the HLE/LE for non-lonely and lonely older adults were 84.2% and 80.1%, respectively (Table 3).

**Table 3:** Gender differences in HLE/LE (%) for lonely and non-lonely older adults by ADL and SRH

	ADL^a^						SRH^b^					
	Non-lonely			Lonely			Non-lonely			Lonely		
	Men	Women	GD^c^	Men	Women	GD^c^	Men	Women	GD^c^	Men	Women	GD^c^
Age												
65	85.0	78.0	6.9	86.4	80.4	6.0	84.2	80.6	3.6	80.1	75.8	4.3
70	81.7	74.0	7.7	83.2	76.4	6.8	84.6	81.1	3.5	80.6	76.4	4.2
75	77.4	68.9	8.5	79.0	71.2	7.8	85.1	81.6	3.5	81.2	76.9	4.2
80	71.5	62.3	9.2	73.3	64.5	8.7	85.6	82.1	3.5	81.8	77.5	4.2
85	63.9	54.2	9.7	65.7	56.2	9.6	86.1	82.7	3.4	82.4	78.2	4.2
90	54.2	44.6	9.6	56.2	46.2	10.0	86.6	83.3	3.4	83.1	78.9	4.2
95	42.9	34.3	8.6	44.8	35.3	9.5	87.2	83.9	3.3	83.7	79.6	4.1
99	33.4	26.4	7.1	35.0	26.8	8.2	87.6	84.4	3.3	84.3	80.2	4.1
AD^d^	51.5	51.7		51.4	53.6		-3.4	-3.7		-4.2	-4.4	

Notes: a. ADL indicates activity of daily living; b. SRH indicates self-rated health; c. GD indicates gender differences, calculated by subtracting the HLE/LE of men from women; and d. AD representing the age decrement in HLE/LE between 65 and 99 years old

From the perspective of gender differences, regardless of whether based on ADL or SRH, the HLE/LE for women were consistently lower than those for men (Fig. 4 and Fig. 5). This indicates that the quality of life for older women is relatively poorer.

The impact of loneliness on health varies depending on the measurement indicators and gender. For example, based on ADL, loneliness had a greater effect on HLE/LE decline for women (Table 3). Specifically, the HLE/LE decline between non-lonely and lonely women from aged 65 to 99 was 51.7% and 53.6%, respectively, resulting in a 1.9% difference, while for men, the impact of loneliness was less significant, with declines of 51.5% and 51.4% for non-lonely and lonely older adults across the older stage, respectively. But according to SRH evaluation, gender differences in the impact of loneliness on HLE/LE decline were not pronounced. In men, the change in HLE/LE for non-lonely older adults was 3.4% throughout the older stage, while for lonely older adults, it was 4.2%, resulting in a difference of 0.8%. For women, the HLE/LE change between non-lonely and lonely older adults during old age was 3.7% and 4.4%, respectively, with a difference of 0.7%.

## Discussion

With the scarcity of research conducted in developing nations, this study employed nationally representative longitudinal data to investigate the influence of loneliness on the health outcomes of older adults in China. Among the lonely population, despite a higher HLE/LE calculated based on ADL, subjective health outcomes are poorer, particularly among females who exhibit relatively lower HLE/LE. The impact of loneliness on health outcomes varies by gender across different measurement indicators, with older women often experiencing a faster decline in HLE/LE based on ADL during the older stage, while the decline in HLE/LE based on SRH tends to be more similar between genders during this stage. This study provides valuable insights into the complex interplay between loneliness and health outcomes, within the context of creating an aging-friendly society.

While lonely older adults may exhibit heightened levels of functional autonomy in ADL, their subjective assessments of health may yield unfavorable outcomes. Specifically, although lonely individuals may potentially achieve higher HLE/LE based on assessments of ADL, they might manifest lower ratios derived from evaluations of SRH. The augmented ADL performance observed among lonely populations may be intimately intertwined with their living arrangements, with solitary domiciles frequently correlating with enhanced physical functionality, as corroborated by extant literature [9]. Nonetheless, in the realm of subjective health, the experience of loneliness can engender heightened psychological distress and emotional isolation, consequently yielding diminished health appraisals. This phenomenon finds resonance across various developed nations [12]. For instance, longitudinal inquiries in Finland have underscored the salience of loneliness as a robust predictor of SRH [19]. Such findings underscore the imperative of not only attending to the functional capacities of lonely individuals but also prioritizing their subjective health to ameliorate their overall well-being and quality of life.

Compared to men, women in the lonely group had a lower HLE/LE, indicating relatively poorer quality of life, as evidenced by both physical functioning and subjective health indicators. Women's health appears to be more susceptible to the adverse effects of loneliness. For example, the Health and Retirement Study showed that chronic loneliness was an independent risk factor for physical functional disability in middle-aged and older women (HR = 1.29, 95% CI = 1.16–1.44). However, there was no significant association with male physical function (HR = 1.13, 95% CI = 0.91–1.40) [21]. This phenomenon can be attributed to differences in biological mechanisms and social attribute. Due to variations in biological mechanisms [34, 35], this often leads to physical discomfort and autoimmune diseases [36, 37]. Furthermore, as a social attribute, gender plays a role in shaping how loneliness is generated and addressed [14]. As the longer LE of older women, coupled with higher chances of experiencing unfavorable life conditions such as widowhood and frequent relocations [20]. Both biological and social mechanisms can amplify feelings of loneliness and contribute to detrimental health effects. Therefore, directed policies should give precedence to the subjective well-being of older women, recognizing their vulnerability, to address health disparities and improve their quality of life.

The impact of loneliness on health outcomes may exhibit gender disparities depending on measurement indicators. Specifically, calculations based on ADL reveal pronounced gender differences in HLE/LE, with a more rapid decline observed among older women during the older stage. This suggests a stronger association between loneliness and ADL among them compared to men. A study conducted in the United States found chronic loneliness to be an independent risk factor for functional disability in middle-aged and older women, while no statistically significant association was observed for men [21]. Conversely, calculations based on subjective health indicate smaller gender differences in the impact of loneliness, possibly due to the comprehensive nature of health measurements. However, research focusing on the psychological health dimension suggests that women's health status may be more susceptible to the influence of loneliness due to longer life expectancy, increased risk of widowhood, and greater susceptibility to adverse health outcomes [38]. This underscores the necessity of considering gender differences across various measurement indicators when evaluating the health of older adults to ensure a comprehensive understanding of their health status.

## Limitations

Several limitations need to be considered. Firstly, despite the high consistency between one-item measure and multi-item scales in assessing loneliness, it may fall short in capturing the multidimensionality and intricacy of loneliness. Secondly, due to model constraints, the inclusion of an extensive number of covariates proves challenging. Subsequent research endeavors should incorporate additional health-related variables, facilitating a more nuanced exploration of the intricate mechanisms underpinning the association between loneliness and diverse dimensions of healthy life expectancy. Thirdly, it is essential to acknowledge an individual's physical functioning and self-rated health status may also influence their sense of loneliness. Therefore, future studies may benefit from research designs or experimental interventions to better elucidate the directionality and mechanisms underlying the observed relationships, thereby providing a more comprehensive understanding of the interplay between health status and loneliness. Nonetheless, these results hold significance for enhancing the subjective well-being of older adults, particularly in developing countries like China undergoing demographic transitions.

## Conclusions

With rapid aging, declining fertility rates, and increased population mobility, more and more older adults are facing living alone, which exacerbates their feelings of loneliness and impacts the health status. This study analyzed the association between loneliness and health among Chinese older adults based on nationally representative data. The results indicate that loneliness has a differential impact on health based on gender, and the effects observed across different health measurement indicators are not entirely consistent. It is crucial to focus on the effects of loneliness on subjective health of older adults, particularly on the quality of life for females, with particular attention to its effects on physical functioning. Therefore, corresponding health promotion measures should target the characteristics of different health indicators and population variances to mitigate the negative health impacts of loneliness, thereby promoting improvements in the health status and subjective well-being of older adults.

## Supplementary Information

Supplementary Material 1: Figure S1. Data quality comparison between the sixth census Data and weighted data. Table S1. Transition probabilities for lonely and non-lonely older adults with standard error by ADL. Table S2. Transition probabilities for lonely and non-lonely older adults with standard error by SRH. Table S3. Population-based LE, HLE and HLE/LE (%) for lonely and non-lonely older adults by gender according to ADL, with 95% confidence intervals. Table S4. Population-based LE, HLE and HLE/LE (%) for lonely and non-lonely older adults by gender according to SRH, with 95% confidence intervals.

## Data Availability

The datasets used and analyzed during the current study are publicly available from Peking University Open Research Data Platform. (https://opendata.pku.edu.cn/dataverse/CHADS?from=timeline&isappinsta).
