# Supplementary material for: Loneliness shapes disparities in healthy life expectancy: a multi-state analysis from China
Source: BMC Public Health. 2024 Jun 4;24:1492. doi: 10.1186/s12889-024-18975-z (PMC11514865; doi:10.1186/s12889-024-18975-z)
Supplement: Supplementary file 1 — Supplementary Material 1: Figure S1. Data quality comparison between the sixth census Data and weighted data. Table S1. Transition probabilities for lonely and non-lonely older adults with standard error by ADL. Table S2. Transition probabilities for lonely and non-lonely older adults with standard error by SRH. Table S3. Population-based LE, HLE and HLE/LE (%) for lonely and non-lonely older adults by gender according to ADL, with 95% confidence intervals. Table S4. Population-based LE, HLE and HLE/LE (%) for lonely and non-lonely older adults by gender according to SRH, with 95% confidence intervals. [file 12889_2024_18975_MOESM1_ESM.docx]

**Supplementary Material**

**Data quality assessment**


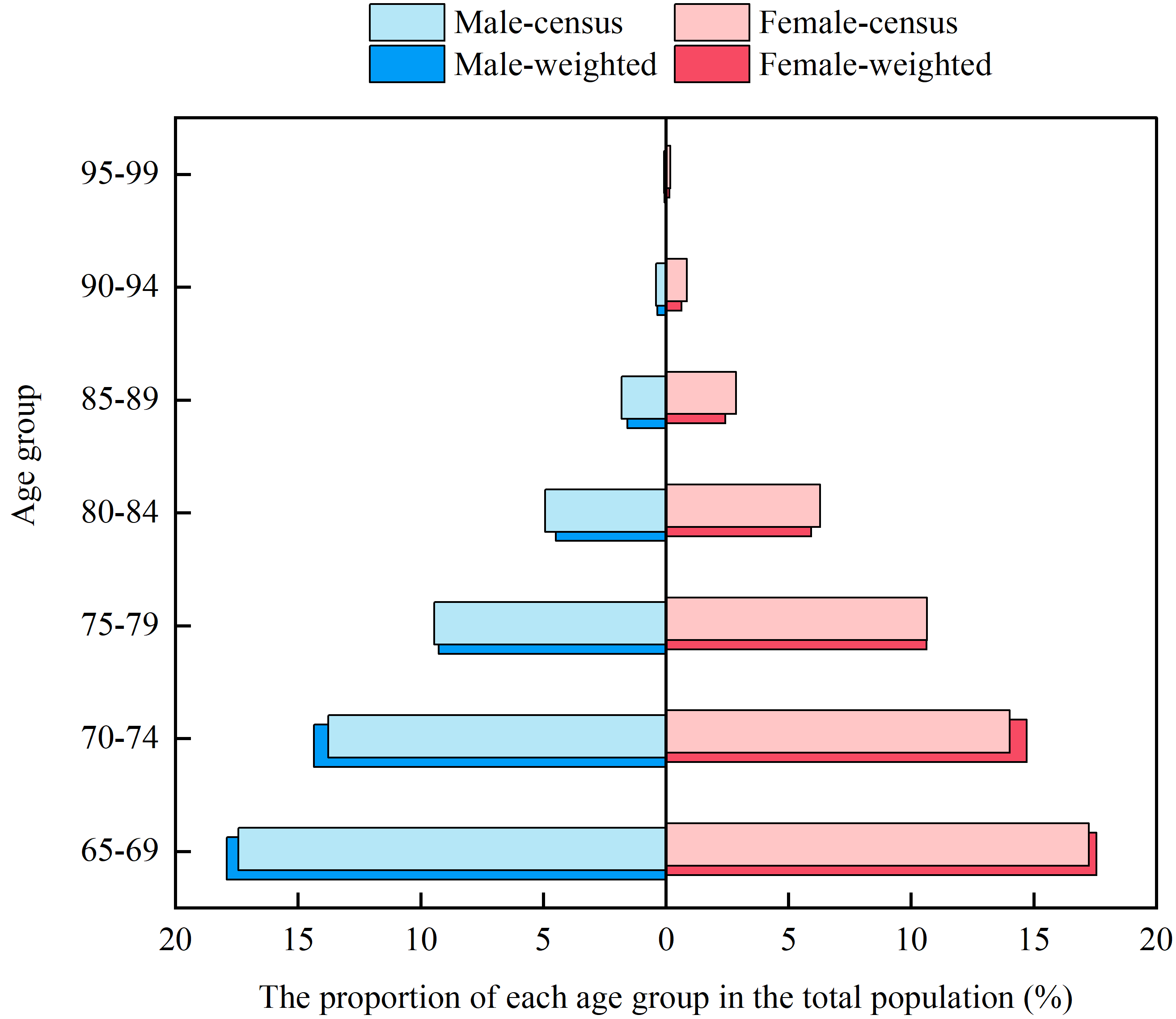


Figure S1 Data quality comparison between the sixth census Data and weighted data

**Transition probabilities between different health states**

Table S1 presents the transition probabilities between different health states calculated based on activities of daily living (ADL) among lonely and non-lonely populations. It can be observed that the transition probabilities leading to unhealthy status and death, namely S2 and S3 (including p_S1→S1_, p_S1→S3_, p_S2→S2_, p_S2→S3_), are higher in the lonely population compared to the non-lonely population. Conversely, the probabilities leading to a healthy outcome, namely S1 (including p_S1→S1_, p_S2→S1_), are higher in the non-lonely population.

| Table S1 Transition probabilities for lonely and non-lonely older adults with standard error by ADL | | | | | | |
| --- | --- | --- | --- | --- | --- | --- |
| Age | p_S1→S1_ | p_S1→S2_ | p_S1→S3_ | p_S2→S1_ | p_S2→S2_ | p_S2→S3_ |
| Lonely |  |  |  |  |  |  |
| 65 | 0.941 (0.003) | 0.034 (0.003) | 0.025 (0.002) | 0.424 (0.039) | 0.436 (0.037) | 0.14 (0.015) |
| 70 | 0.913 (0.004) | 0.05 (0.003) | 0.037 (0.002) | 0.348 (0.027) | 0.483 (0.027) | 0.17 (0.014) |
| 75 | 0.873 (0.004) | 0.073 (0.004) | 0.055 (0.002) | 0.278 (0.019) | 0.521 (0.02) | 0.201 (0.011) |
| 80 | 0.818 (0.005) | 0.103 (0.005) | 0.08 (0.003) | 0.217 (0.016) | 0.55 (0.018) | 0.233 (0.01) |
| 85 | 0.746 (0.008) | 0.142 (0.007) | 0.113 (0.005) | 0.166 (0.017) | 0.57 (0.019) | 0.264 (0.011) |
| 90 | 0.658 (0.012) | 0.188 (0.011) | 0.154 (0.007) | 0.125 (0.018) | 0.58 (0.024) | 0.295 (0.016) |
| 95 | 0.557 (0.016) | 0.241 (0.016) | 0.202 (0.012) | 0.093 (0.017) | 0.582 (0.03) | 0.325 (0.024) |
| 99 | 0.473 (0.019) | 0.284 (0.021) | 0.243 (0.016) | 0.073 (0.016) | 0.58 (0.036) | 0.348 (0.031) |
| Non-lonely | | | | | | |
| 65 | 0.951 (0.002) | 0.029 (0.002) | 0.02 (0.001) | 0.518 (0.04) | 0.41 (0.038) | 0.073 (0.009) |
| 70 | 0.927 (0.002) | 0.043 (0.002) | 0.03 (0.001) | 0.439 (0.028) | 0.47 (0.027) | 0.091 (0.008) |
| 75 | 0.892 (0.002) | 0.063 (0.002) | 0.045 (0.001) | 0.363 (0.019) | 0.525 (0.019) | 0.112 (0.008) |
| 80 | 0.844 (0.003) | 0.089 (0.003) | 0.066 (0.002) | 0.293 (0.016) | 0.573 (0.017) | 0.134 (0.008) |
| 85 | 0.78 (0.006) | 0.125 (0.005) | 0.095 (0.003) | 0.232 (0.019) | 0.611 (0.02) | 0.157 (0.009) |
| 90 | 0.699 (0.01) | 0.169 (0.009) | 0.132 (0.006) | 0.18 (0.022) | 0.64 (0.025) | 0.18 (0.013) |
| 95 | 0.604 (0.015) | 0.22 (0.015) | 0.177 (0.01) | 0.137 (0.023) | 0.66 (0.031) | 0.204 (0.019) |
| 99 | 0.52 (0.019) | 0.264 (0.02) | 0.216 (0.015) | 0.109 (0.023) | 0.669 (0.036) | 0.222 (0.025) |
| Notes: p_S1→S1_ indicates the transition probabilities from healthy to healthy; p_S1→S2_ indicates the transition probabilities from healthy to unhealthy; p_S1→S3_ indicates the transition probabilities from healthy to death; p_S2→S1_ indicates the transition probabilities from unhealthy to healthy; p_S2→S2_ indicates the transition probabilities from unhealthy to unhealthy; p_S2→S3_ indicates the transition probabilities from unhealthy to death. | | | | | | |

Table S2 displays the transition probabilities between different health states based on self-rated health (SRH), both among lonely and non-lonely older adults. We have observed similarities between Table S1 and Table S2. Compared to the non-lonely population, the transition probabilities leading to unhealthy outcomes and status, namely p_S1→S1_, p_S1→S3_, p_S2→S2_, p_S2→S3_, were consistently higher among lonely older adults. However, the non-lonely population exhibited higher probabilities of transitioning to a healthy outcome, specifically in terms of p_S1→S1_ and p_S2→S1_.

| Table S2 Transition probabilities for lonely and non-lonely older adults with standard error by SRH | | | | | | |
| --- | --- | --- | --- | --- | --- | --- |
| Age | p_S1→S1_ | p_S1→S2_ | p_S1→S3_ | p_S2→S1_ | p_S2→S2_ | p_S2→S3_ |
| Lonely | | | | | | |
| 65 | 0.82 (0.01) | 0.156 (0.01) | 0.024 (0.002) | 0.474 (0.027) | 0.473 (0.027) | 0.054 (0.006) |
| 70 | 0.814 (0.008) | 0.15 (0.008) | 0.036 (0.002) | 0.466 (0.02) | 0.458 (0.02) | 0.077 (0.006) |
| 75 | 0.802 (0.007) | 0.144 (0.006) | 0.054 (0.003) | 0.453 (0.016) | 0.439 (0.016) | 0.109 (0.007) |
| 80 | 0.783 (0.007) | 0.137 (0.006) | 0.08 (0.003) | 0.434 (0.016) | 0.414 (0.017) | 0.151 (0.008) |
| 85 | 0.754 (0.009) | 0.128 (0.007) | 0.118 (0.005) | 0.409 (0.021) | 0.384 (0.021) | 0.207 (0.012) |
| 90 | 0.712 (0.011) | 0.117 (0.008) | 0.171 (0.008) | 0.376 (0.026) | 0.348 (0.026) | 0.276 (0.02) |
| 95 | 0.655 (0.014) | 0.105 (0.009) | 0.24 (0.013) | 0.336 (0.03) | 0.306 (0.031) | 0.358 (0.029) |
| 99 | 0.598 (0.018) | 0.094 (0.01) | 0.308 (0.017) | 0.299 (0.033) | 0.27 (0.034) | 0.431 (0.037) |
| Non-lonely | | | | | | |
| 65 | 0.856 (0.006) | 0.126 (0.006) | 0.018 (0.001) | 0.511 (0.023) | 0.44 (0.023) | 0.049 (0.005) |
| 70 | 0.851 (0.004) | 0.121 (0.004) | 0.028 (0.001) | 0.503 (0.016) | 0.426 (0.016) | 0.07 (0.005) |
| 75 | 0.841 (0.003) | 0.117 (0.003) | 0.042 (0.001) | 0.491 (0.013) | 0.41 (0.013) | 0.1 (0.005) |
| 80 | 0.826 (0.004) | 0.111 (0.004) | 0.063 (0.002) | 0.472 (0.016) | 0.389 (0.015) | 0.14 (0.007) |
| 85 | 0.802 (0.006) | 0.105 (0.005) | 0.094 (0.003) | 0.446 (0.021) | 0.362 (0.021) | 0.192 (0.012) |
| 90 | 0.766 (0.008) | 0.097 (0.007) | 0.137 (0.006) | 0.413 (0.027) | 0.33 (0.027) | 0.257 (0.02) |
| 95 | 0.716 (0.012) | 0.089 (0.008) | 0.195 (0.01) | 0.371 (0.033) | 0.292 (0.032) | 0.336 (0.03) |
| 99 | 0.665 (0.016) | 0.08 (0.008) | 0.255 (0.015) | 0.334 (0.037) | 0.26 (0.035) | 0.407 (0.039) |
| Notes: p_S1→S1_ indicates the transition probabilities from healthy to healthy; p_S1→S2_ indicates the transition probabilities from healthy to unhealthy; p_S1→S3_ indicates the transition probabilities from healthy to death; p_S2→S1_ indicates the transition probabilities from unhealthy to healthy; p_S2→S2_ indicates the transition probabilities from unhealthy to unhealthy; p_S2→S3_ indicates the transition probabilities from unhealthy to death. | | | | | | |

**Sensitive analysis**

Table S3 and Table S4 present the results of life expectancy (LE), healthy life expectancy (HLE), and the proportion of healthy life expectancy in life expectancy (HLE/LE) for lonely and non-lonely older adults by gender, based on ADL and SRH. The results demonstrate that, regardless of gender or the measure used (ADL or SRH), lonely older adults exhibit worse LE and HLE, compared to their non-lonely counterparts. As for HLE/LE, the lonely population had a higher proportion of remaining life expectancy attributed to independent living survival time, but a lower proportion attributed to self-rated health survival time.

| Table S3 Population-based LE, HLE and HLE/LE (%) for lonely and non-lonely older adults by gender according to ADL, with 95% confidence intervals | | | | | | |
| --- | --- | --- | --- | --- | --- | --- |
| Age | LE | HLE | HLE/LE | LE | HLE | HLE/LE |
|  | Non-lonely | | | Lonely | | |
| Males | | | | | | |
| 65 | 20.6(20.1,21.2) | 17.5(17.1,18) | 85.0 | 17.4(16.7,18) | 15(14.4,15.6) | 86.4 |
| 75 | 13.9(13.4,14.5) | 10.8(10.4,11.2) | 77.4 | 11.2(10.7,11.6) | 8.8(8.4,9.3) | 79.0 |
| 85 | 9(8.4,9.6) | 5.8(5.3,6.2) | 63.9 | 6.9(6.5,7.3) | 4.5(4.1,4.9) | 65.7 |
| Females | | | | | | |
| 65 | 25.6(24.7,26.4) | 19.9(19.4,20.5) | 78.0 | 21.3(20.6,21.9) | 17.1(16.5,17.7) | 80.4 |
| 75 | 18.2(17.3,19.1) | 12.5(12,13.1) | 68.9 | 14.4(13.8,14.9) | 10.2(9.7,10.7) | 76.4 |
| 85 | 12.5(11.5,13.6) | 6.8(6.2,7.3) | 54.2 | 9.3(8.7,9.9) | 5.2(4.8,5.7) | 56.2 |

| Table S4 Population-based LE, HLE and HLE/LE (%) for lonely and non-lonely older adults by gender according to SRH, with 95% confidence intervals | | | | | | |
| --- | --- | --- | --- | --- | --- | --- |
| Age | LE | HLE | HLE/LE | LE | HLE | HLE/LE |
|  | Non-lonely | | | Lonely | | |
| Males | | | | | | |
| 65 | 20.2(19.7,20.7) | 17(16.5,17.5) | 84.2 | 17.7(17,18.3) | 14.1(13.5,14.8) | 80.1 |
| 75 | 13.6(13.2,14.1) | 11.6(11.1,12) | 85.1 | 11.6(11.1,12.1) | 9.4(8.9,9.9) | 81.2 |
| 85 | 8.6(8.1,9) | 7.4(6.9,7.8) | 86.1 | 7.1(6.7,7.6) | 5.9(5.5,6.3) | 82.4 |
| Females | | | | | | |
| 65 | 24.4(23.8,25.1) | 19.7(19.1,20.3) | 80.6 | 21.8(21.1,22.4) | 16.5(15.8,17.2) | 75.8 |
| 75 | 17.1(16.5,17.7) | 14(13.4,14.5) | 81.6 | 14.9(14.3,15.4) | 11.4(10.9,12) | 76.9 |
| 85 | 11.2(10.6,11.7) | 9.2(8.6,9.8) | 82.7 | 9.5(8.9,10) | 7.4(6.9,7.9) | 78.2 |
